# Laser-Assisted Wafering of 4H-SiC: From Subsurface Modification and Crack Coalescence to Controlled Separation

**DOI:** 10.3390/ma19214688

**Published:** 2026-11-03

**Authors:** Zhiqiang Fan, Pengsen Zhang, Beichen Hu

**Affiliations:** 1 School of Mechanics and Transportation Engineering, Northwestern Polytechnical University, Xi’an 710072, China; zps@mail.nwpu.edu.cn; 2 The Second Research Institute of China Electronics Technology Group Corporation, Taiyuan 030024, China; beichen.hu@foxmail.com

**Keywords:** 4H-SiC, laser wafering, subsurface modification, crack coalescence, fracture mechanics, wafer separation, ultrasonic assistance, cold splitting

## Abstract

**Highlights:**

**What are the main findings?**

- Laser wafering is governed by crack-network connectivity, not laser damage alone.
- Reported separation strengths range from 38 MPa to about 0.04 MPa.
- As-split roughness is controlled mainly by laser processing and the 4° off-cut.

**What are the implications of the main findings?**

- Modification and separation should be optimized as one coupled process.
- Standardized stripping tests are needed for meaningful cross-study comparison.

**Abstract:**

Laser-assisted wafering can reduce material consumption in silicon carbide (SiC) substrate manufacturing, but successful subsurface modification does not, by itself, guarantee controlled wafer release. This review treats 4H-SiC wafering as a two-step process comprising laser formation of a weakened subsurface layer, followed by assisted separation. We connect nonlinear energy deposition, phase transformation and residual stress to crack nucleation, interaction and coalescence, and then assess mechanical and ultrasonic separation, polymer-assisted cold splitting, continuous-wave laser splitting, other thermal-stress routes, and chemical or hybrid release within a common fracture-mechanics framework. Reported results show that crystallographic orientation, scan arrangement, crack coplanarity, acoustic coupling and liquid medium strongly affect separation. Nominal separation stress, residual stripping force and internally generated thermal stress are distinct metrics and should not be compared directly. Reported separation strengths span more than three orders of magnitude, from above 38 MPa to about 0.04 MPa, primarily because laser processing produces very different crack-network connectivity. As-split roughness ranges from about 0.16 to 6.5 µm and is governed mainly by the laser step and 4° off-cut geometry. Joint optimization of modification and separation, together with standardized reporting, is therefore essential for scalable low-loss wafering.

## 1. Introduction

### 1.1. SiC and the Substrate Cost Problem

4H-SiC is the substrate of choice for high-voltage, high-temperature and high-frequency power devices. Its advantages are a wide bandgap (about 3.2–3.26 eV), a high critical field (about 3 MV/cm) and high thermal conductivity [1,2]. Semi-insulating 4H-SiC is also closely lattice-matched to GaN, which makes it the preferred substrate for GaN-on-SiC RF devices [3]. Adoption is nonetheless constrained by cost. He et al. [2] estimate that the substrate accounts for roughly half of SiC device cost. SiC crystal growth is slow and energy-intensive, making the grown ingot a major contributor to substrate manufacturing costs. Increasing the number of usable wafers recovered from each ingot distributes these upstream costs across a larger output and therefore reduces the growth-related cost per wafer. Minimizing kerf loss, subsurface damage and the material removed during subsequent finishing is thus essential to improving material utilization, provided that wafer quality and production yield are maintained.

### 1.2. Limits of Wire Sawing

SiC ingots are sliced mainly by multi-wire sawing with loose slurry or fixed diamond abrasives [4,5]. Because of the hardness (Mohs 9.2–9.5) and brittleness of SiC, the process is slow and wasteful. Kerf losses of about 150–250 µm per cut are commonly reported [1,2,6,7]. Slurry sawing of a 6-inch boule can take more than 100 h [7]. Sawn wafers carry roughness, subsurface damage and warp, which must be removed by lapping, grinding and chemical–mechanical polishing. Diamond-wire wafers may also require high-temperature annealing to relieve warpage [7]. Across the full chain, 30–50% of the grown crystal can be lost [2,8]. Kaule et al. [9] estimated that producing a 350 µm SiC wafer consumes about 300 µm of additional material conventionally, against about 100 µm for a kerf-less spalling route. This corresponds to a yield gain of up to one third. Vertical devices are, moreover, thinned by 80% or more after front-side processing, which motivates starting from thinner wafers [10]. As wafer diameter grows to 200 mm and beyond, wire tension and cutting forces become harder to control [3,11].

### 1.3. Laser Modification and Subsequent Separation

**Figure 1:**
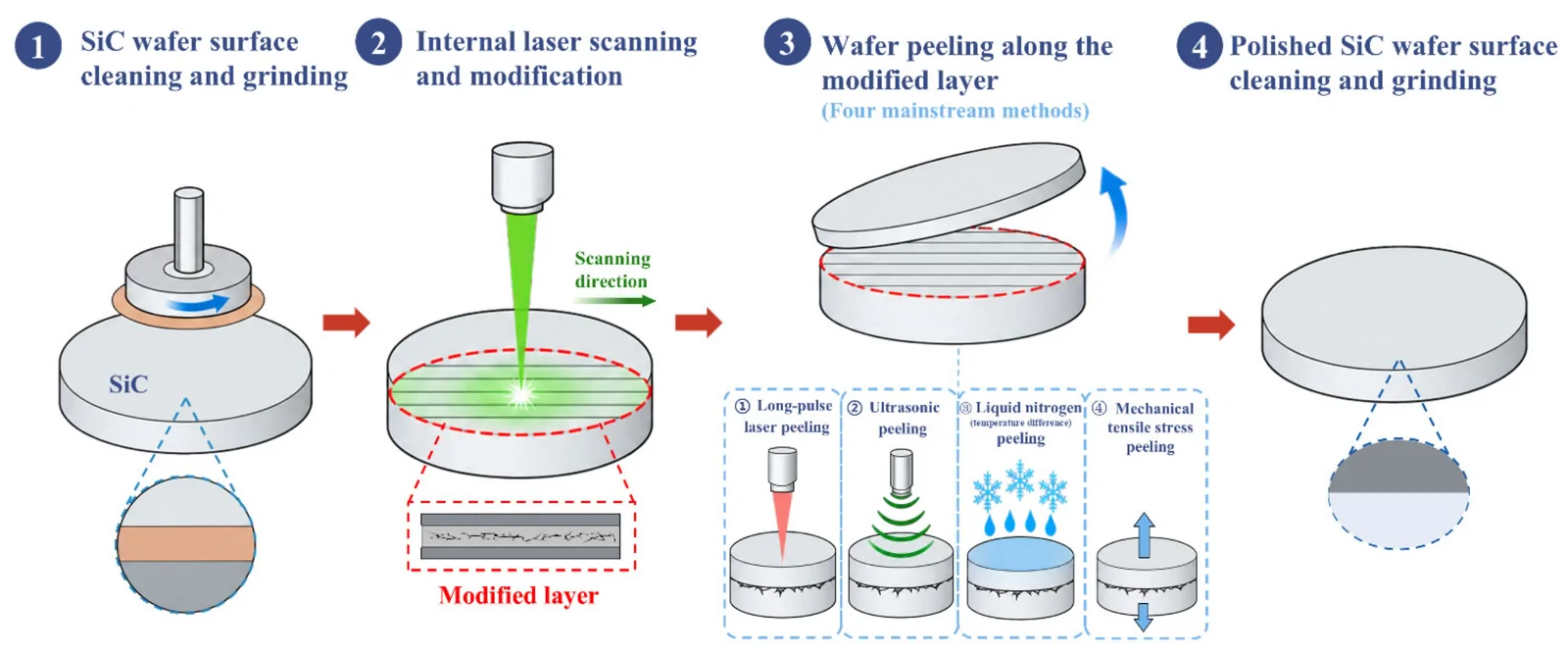
A schematic of laser modification and subsequent separation for SiC wafering. Subsurface laser modification creates modified regions and microcracks at depth, and then assisted separation (mechanical, ultrasonic, cold split, CW laser, thermal, chemical or hybrid) releases the wafer. The wafer is then finished, and the remaining ingot is resurfaced and reprocessed.

**Figure 2:**
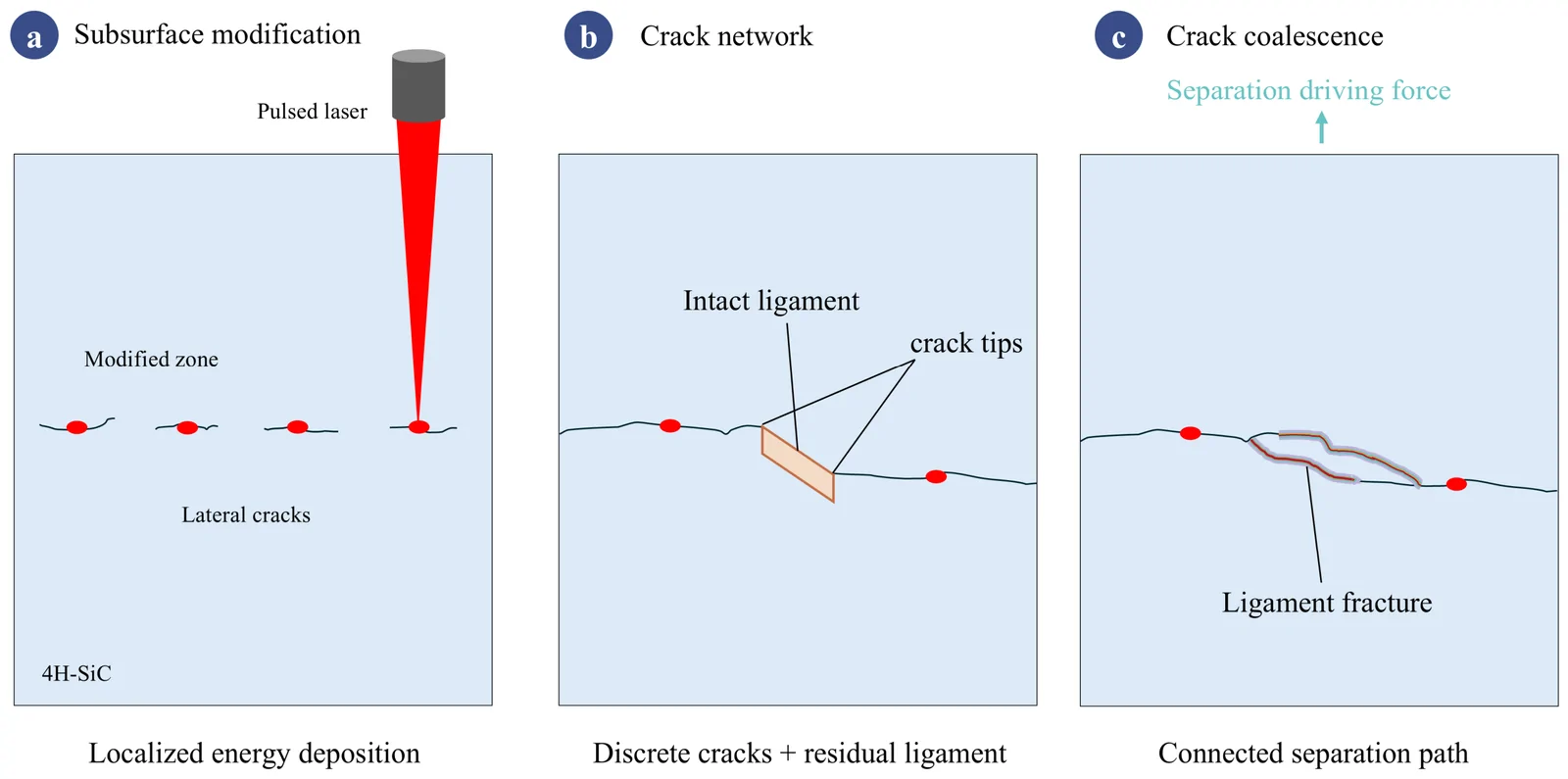
Fracture evolution linking laser modification to wafer separation. (**a**) Pulsed-laser irradiation creates discrete modified zones and lateral microcracks. (**b**) Neighboring cracks remain partly connected by intact ligaments; crack-tip spacing, vertical offset, coplanarity and ligament geometry therefore control the residual load-bearing area. (**c**) Under a separation-driving force, the cracks extend, turn and coalesce through ligament rupture to form a continuous release path. Reliable wafering thus requires a connected, depth-confined crack network rather than modified tracks alone.

Laser modification of 4H-SiC uses a focused pulsed laser to induce localized structural changes and microcracks beneath the surface, creating a weakened layer that facilitates subsequent wafer separation [3,6,12,13]. Following subsurface laser modification, wafer separation is achieved by extending and connecting the induced cracks through mechanical loading, ultrasonic assistance, polymer-assisted cold splitting, CW-laser heating or other thermal-stress treatments, with chemical or hybrid methods providing additional routes to release [7,8,10,14,15] (Figure 1). The central difficulty is that a laser-written region is not necessarily a continuous fracture plane. Discrete modified tracks can be linked by lateral cracks yet remain connected through intact ligaments, depth offsets or residual material within the tracks [3,14,16] (Figure 2). Changing scan direction, processing sequence or line spacing can consequently alter the separation load without changing the nominal focal depth [1,3]. A useful description must resolve both the directly modified material and the surrounding crack network.

Two kerf-less concepts provide important precedents for this route. Controlled spalling uses a stressed surface layer to drive a crack approximately parallel to the free surface; the steady propagation depth is governed by elastic mismatch, stressor thickness and mode mixity, rather than by a saw kerf [17,18,19]. Stealth dicing instead focuses a sub-bandgap laser inside a transparent crystal to create buried damage tracks that are connected by a small subsequent load [20]. Two-step SiC wafering combines these ideas: the laser defines the target release depth, while a mechanically, acoustically, thermally or chemically generated driving field completes fracture along the prepared plane.

Industrial implementations illustrate the same separation of functions. Siltectra’s COLD SPLIT process uses subsurface laser conditioning, followed by contraction of a bonded polymer stressor, whereas KABRA-type routes use laser modification, followed by mechanical separation, with ultrasonic assistance available in related process variants [8,9,10,21]. These examples motivate treating laser modification and wafer release as a coupled design problem rather than optimizing either step in isolation.

Several recent reviews cover laser–SiC interaction and pulsed-laser machining, including drilling, grooving, dicing and slicing [22,23]. Others cover control of modified-layer thickness [12] and the state of laser-based exfoliation [21]. In these accounts, separation is typically a final processing step. Yet the laser step alone does not produce a wafer. A modified ingot retains a bonding strength of about 1 MPa to more than 38 MPa, depending on laser conditions and crystal orientation [1,6]. How this residual bonding is overcome controls roughness, damage depth, handling risk and cycle time.

This review is therefore organized around the separation step, using fracture mechanics as a common bridge. Section 2 examines two-step laser-assisted wafering from subsurface energy deposition to formation of a separable crack network and its coupling to release. Section 3 reviews mechanical and ultrasonic separation, cold splitting, thermal-stress-driven approaches, and chemical or hybrid routes. Section 4 compares separation performance, surface integrity, material utilization and manufacturing challenges. Section 5 provides the conclusions and outlook.

## 2. Two-Step Laser-Assisted Wafering

### 2.1. Subsurface Energy Deposition and Material Modification

Internal processing requires sufficient optical transmission through the overlying SiC and localized absorption near the intended depth. Under suitable irradiation conditions, nonlinear excitation produces a dense electronic population, followed by structural transformation, stress generation and cracking. The response depends on wavelength, pulse duration, energy, focusing and the optical properties of the starting crystal. It also depends on irradiation history, because previously modified material can absorb subsequent pulses differently from intact SiC [3,6,13].

Nonlinear propagation can substantially reshape the focal volume. In 4H-SiC stealth dicing, Wang et al. linked multiple axial modification sites to self-focusing and refocusing [24]. Their femtosecond–picosecond comparison associated femtosecond exposure with nanovoids and microcracks, while picosecond exposure produced larger modified regions and stronger thermal ablation at matched fluence [25]. Xie et al. further showed that scan speed changed the number and arrangement of internal layers; easier fracture under a multilayer condition was accompanied by poorer cross-sectional integrity [26]. These through-thickness experiments establish a modification–damage trade-off, although their elongated axial structures are not the target geometry for horizontal wafer release.

Focal depth alone does not specify the resulting damage distribution. Tian et al. used spatial light-modulator phase correction to compensate for refractive-index mismatch during 1030 nm, 10 ps processing of semi-insulating 4H-SiC [27]. They distinguished a primary upper region associated with plasma-enhanced absorption from an unwanted lower region near the actual optical focus. Phase correction shortened the axial damage spot from approximately 13.5 to 7.2 µm in a low-energy comparison. Subsequent optimization of pulse energy and scan spacing suppressed the lower modified region while retaining lateral crack connectivity. The useful control target is therefore the depth distribution of energy deposition and damage. In silicon stealth dicing, Qiao et al. additionally showed that the spatial distribution of phase-wrap transition regions on the modulator affects focal energy concentration [28]. This optical-design result is relevant to correction strategies.

At the near-infrared wavelengths most often used for internal modification, 4H-SiC remains sufficiently transparent for energy to reach the interior, while the focal intensity drives nonlinear absorption. Reported transmission at 1028–1064 nm is about 65–68%, including surface reflection, and a 1064 nm photon carries about 1.16 eV, so approximately three photons are required to bridge the 4H-SiC bandgap [13,20]. Because the multiphoton rate rises steeply with intensity, energy deposition can be restricted to a micrometer-scale focal volume. At wafering depths of roughly 100–300 μm, however, refractive-index mismatch produces spherical aberration and focal displacement; spatial light modulator (SLM)-based phase correction and depth-dependent power compensation are therefore important for maintaining a narrow and uniform modification plane [1,6,27,28].

The modified zone is not a single homogeneous phase. Raman, TEM and diffraction observations across femtosecond, picosecond and sub-nanosecond processing identify amorphous silicon, amorphous or graphitic carbon, polycrystalline material, voids and, in some cases, 3C-SiC [3,6,13,14]. Decomposition and re-solidification produce local eigenstrains and residual stress. These fields promote mode-I microcrack nucleation, while the new phases also alter optical absorption and chemical reactivity. Consequently, the same laser-written layer simultaneously acts as a weakened mechanical interlayer, an absorber for later CW heating and a selectively removable region for chemical or photoelectrochemical release [7,15].

Pulse duration changes the geometry and connectivity of this structure. Femtosecond double pulses can suppress the strong axial elongation observed for single ultrashort pulses and broaden the processing window [6]. Picosecond micro-explosion readily forms separable layers at high scan speed [13], whereas overlapping sub-nanosecond pulses can exploit absorption by previously formed graphite and silicon to support self-propagating crack growth [3]. The useful comparison between laser families is therefore not pulse width alone; it is also the combined damage thickness, lateral crack reach, track spacing and writing speed required to create a connected release plane.

Microscopy and spectroscopy show that the resulting layer is chemically and structurally heterogeneous. Kim et al. identified amorphization and decomposition into silicon- and carbon-containing products after femtosecond double-pulse processing [6]. Han et al. associated picosecond-induced micro-explosions with amorphous silicon and carbon, reporting a directly modified width below approximately 2 µm under selected conditions [13]. Wang et al. identified graphite and amorphous silicon in a thin damage layer produced using their nominally sub-nanosecond system [3]. Li et al., by contrast, described an amorphous and 3C-SiC-containing region after nanosecond modification [14]. These observations establish that a single composition or constitutive law cannot represent every laser-modified layer.

Structural transformation serves several functions. It can reduce local fracture resistance, generate eigenstrains that nucleate cracks, increase absorption during subsequent irradiation and provide chemical selectivity for removal. The importance of each function depends on the intended separation route. For example, absorption contrast is central to sequential CW-laser splitting, whereas phase selectivity and liquid access become decisive in chemical release [7,15]. Modification should therefore be designed for the subsequent operation rather than optimized solely for the smallest visible track.

Pulse duration is similarly an incomplete basis for ranking processes. Wu et al. modeled nanosecond slicing by coupling energy deposition, carrier evolution and thermomechanical response, identifying contributions from rapid volume-expansion loading and, later, thermal stress-driven crack growth [29]. Yu et al. demonstrated a multiple-line overlapping strategy for large 4H-SiC crystals [30]. These studies show how scanning and repeated exposure can be used to develop a separable structure with longer pulses. Bessel-beam experiments on SiC also found more continuous internal modification when pulse duration increased from the femtosecond into the picosecond range [31]. The extended axial focus is useful for die singulation, whereas wafer slicing requires confinement around the release depth. Comparisons between laser families must therefore account for crack geometry, damage depth and processing rate, rather than assuming that shorter pulses invariably minimize total material loss.

### 2.2. Formation of a Separable Crack Network

The useful fracture network extends beyond the directly modified tracks. Lateral crack growth reduces the intact distance between neighboring tracks and can lower the number of scans needed for release. Excessive out-of-plane growth, however, increases surface relief and the depth that must be removed subsequently. Wafering therefore requires connected cracks concentrated within a narrow depth range, not simply maximum crack length [3,13].

Line interval is especially important because connectivity is non-monotonic. If tracks are too widely spaced, cracks terminate before reaching neighboring tracks, and intact ligaments dominate the separation load. If spacing is too small, excessive overlap can enlarge the transformed volume, increase optical shielding and promote fragmentation. Representative optima reported for different regimes range from a range of about 6–10 μm for picosecond micro-explosion to a range of 20–25 μm for femtosecond writing and about 100 μm for sub-nanosecond self-propagating cracking [3,6,13]. These values are process-specific because the relevant length scale is the lateral crack-tip interaction distance, not the programmed pitch by itself.

Crystallography influences both nucleation and propagation. Directional fracture and tensile measurements confirm anisotropic behavior in single-crystal SiC [32]. Nanoindentation studies by Liu et al. and Fan et al. reported lower fracture resistance along $\left[1\;\bar{1}\;0\;0\right]$ than along $\left[1\;1\;\bar{2}\;0\right]$, with higher resistance on the Si face than on the C face [33,34]. Hou et al. showed that inferred toughness also depends on indenter geometry, load and the accompanying radial or lateral crack system [35]. The measurements inform anisotropic models but do not directly specify the resistance of laser-modified material.

During slicing, crystallography additionally interacts with incident face, off-axis orientation and the spatial sequence of energy deposition. Yao et al. compared semi-insulating and n-type 4H-SiC under picosecond processing and found favorable separation for C-face incidence with scanning along $\left[1\;\bar{1}\;0\;0\right]$ [11]. Youn et al. showed that scan direction and processing sequence changed crack patterns and separation response even at an unchanged programmed line interval [1]. The scan direction, line-stepping direction and off-axis orientation should consequently be specified independently. For through-thickness dicing, Wen et al. instead measured lower three-point-bending fracture loads after scanning along $\left[1\;1\;\bar{2}\;0\right]$ than along $\left[1\;\bar{1}\;0\;0\right]$ [36]. This does not define a conflicting universal toughness ranking: the modified geometry, fracture plane and loading differ from indentation and horizontal slicing. A preferred scan direction must be established for the complete process configuration.

The separation response can therefore vary by more than an order of magnitude even at nominally unchanged laser settings. Youn et al. reported values from roughly 4.2 MPa to above a 38 MPa instrument limit as scan direction and processing sequence were changed [1]. Yao et al. found the lowest resistance for C-face incidence with scanning along the favorable in-plane direction, and they also reported that n-type material required substantially higher stress than semi-insulating material under matched conditions [11]. These observations reinforce the need to specify incident face, scan direction, line-stepping direction, doping and off-axis angle independently.

Wang et al. directly examined this coupling in 4° off-axis n-type 4H-SiC [16]. Changing the feed direction relative to the inclined basal planes altered crack interference, damage depth and surface morphology. With their selected scan/feed strategy, a 150 µm pitch produced a separation stress of 0.88 MPa. Wider spacing left disconnected cracks, whereas excessive overlap increased the fraction of modified material and promoted fragmentation. Thus, closer tracks did not monotonically improve separation. Their phase-field simulations illustrated crack deflection and linkage between neighboring inclined cracks, providing a mechanistic interpretation of the pitch dependence.

Connectivity is a three-dimensional property. Adjacent cracks can overlap in projection while remaining vertically separated. Their interaction is then governed by crack length, horizontal tip separation, vertical offset and the shape of the intervening ligament. Extension may require crack turning, rupture of a bridge or formation of a stepped connection. Wang et al. linked separation strength to the cracked-area fraction and crack-depth uniformity, while Li et al. identified staggered cracks as an obstacle to complete interconnection [3,14]. These results support using ligament geometry and depth variation alongside average crack length when characterizing a modified specimen.

Beam timing provides another means of controlling this state. Xie et al. [37] compared synchronous dual-beam processing with asynchronous single-beam scanning. At a spacing of 60 µm, the reported peak peeling tensile stress decreased from 6.281 to 4.116 MPa; at 100 µm, it decreased from 2.952 to 2.738 MPa. The corresponding reductions, calculated from the reported values, are 34.5% and 7.2%. The spacing dependence is instructive: the benefit of simultaneous irradiation depends on the interaction between neighboring modified regions. It cannot be described by a universal reduction factor or equated with completion of the later separation step.

### 2.3. Coupling Modification to the Separation Step

For fracture-dominated release, separation occurs when the local driving force is sufficient to extend cracks through the remaining load-bearing regions. Energy-release-rate and stress-intensity descriptions provide the mechanical basis, but the relevant resistance is generally orientation-dependent and spatially variable. Classical analyses of cracking beneath stressed films demonstrate how the loading configuration controls both propagation and the preferred fracture depth [17]. In laser-modified SiC, the crack population and altered material add further spatial variation to that problem.

A useful mesoscale idealization is to regard the prepared plane as a ligament-bridged interlayer. Pre-existing microcracks carry little normal traction, while the intact bridges and incompletely transformed regions carry most of the residual load. The measured nominal separation stress then depends primarily on the remaining load-bearing area, the local bridge strength and the degree to which neighboring cracks are coplanar. This interpretation explains why improved crack connectivity can reduce the apparent separation strength by orders of magnitude without changing the external loading method [3,14,16].

Crack-path control is equally important. Normal tension is comparatively path-neutral and mainly opens the prepared plane, whereas peel, shear, stressor layers and nonuniform thermal fields introduce mixed-mode loading. For stressor-driven spalling, the crack tends toward a depth at which the mode-II stress intensity vanishes; a laser-written weak plane must therefore be strong enough geometrically to capture that crack at the desired depth [17,18,19]. In 4° off-axis 4H-SiC, basal-plane segments are inclined to the nominal wafer plane, so coalescence may require crack turning or rupture of stepped ligaments, which increases both separation load and surface relief [1,14,16].

A frequently reported experimental measure is the nominal separation stress,

*σ_s_* = *F*_max_/*A* (1)

where *F*max is the peak measured force, and *A* is the nominal loaded area. This quantity characterizes the response of a specimen and test arrangement; it is not an intrinsic tensile strength or fracture toughness. Crack connectivity, adhesive compliance, load alignment, bending and the position of final failure all affect its interpretation. A force obtained through a lever-based stripping fixture also requires a clear definition of the measured force and lever geometry before conversion to an area-normalized stress.

Separation treatments can act on different parts of this mechanical problem. External tension and thermal mismatch increase crack driving force. Ultrasound adds time-dependent loading and, in liquids, fluid-mediated effects. Chemical treatment removes or weakens susceptible material and can change the accessibility of buried cracks [7,8,15]. The two-step framework thus permits several operations within each stage, while preserving a clear design question: which modified structure can be released by the selected treatment with limited damage and finishing demand? Representative laser-modification conditions and separation outcomes are summarized in Table 1.

## 3. Separation Strategies

### 3.1. Mechanical and Ultrasonic-Assisted Separation

**Figure 3:**
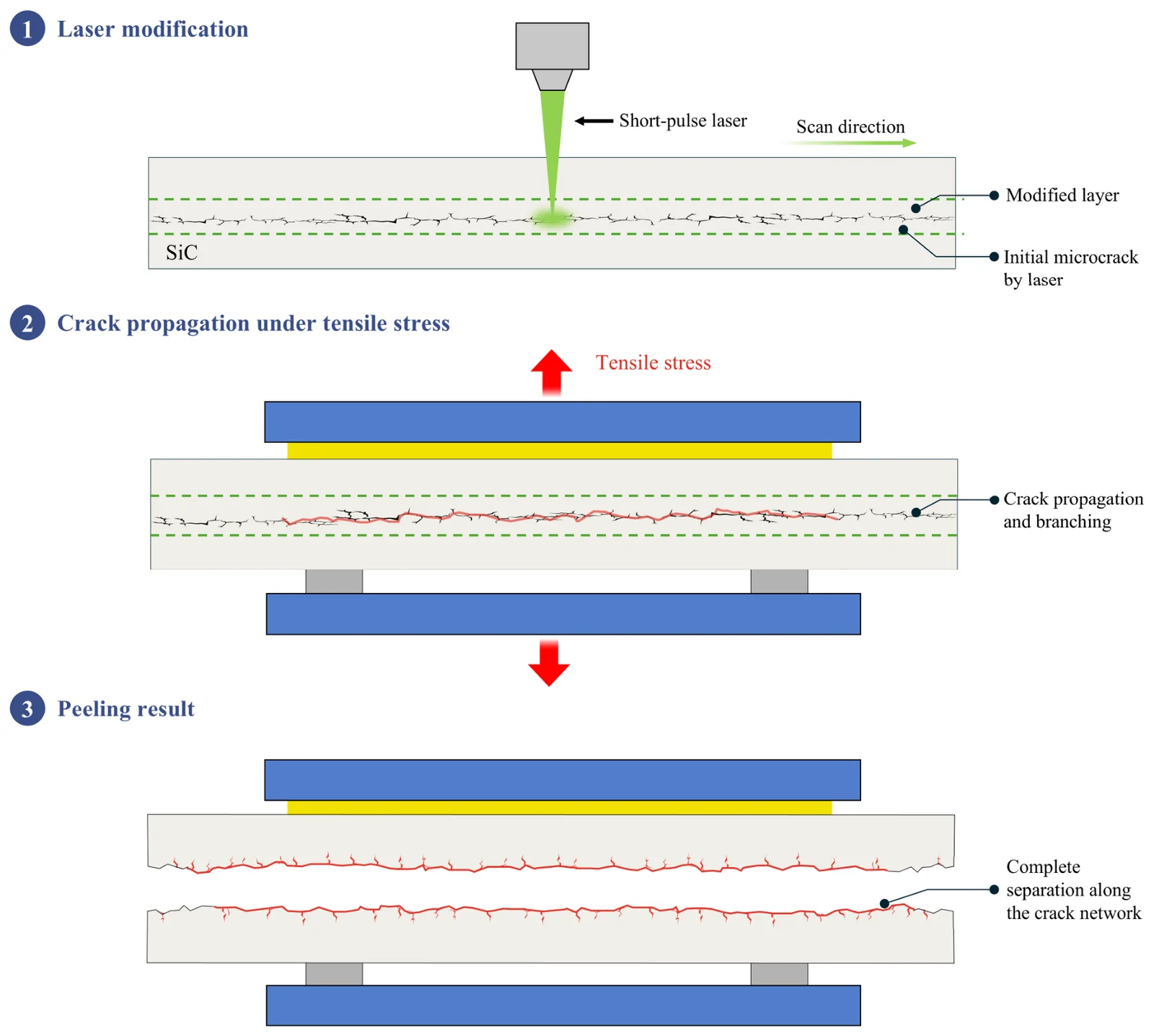
Direct mechanical separation after subsurface laser modification. (**1**) Short-pulse scanning creates a weakened layer containing modified material and initial microcracks. (**2**) Tensile loading normal to this layer opens and extends the prepared cracks while rupturing residual ligaments; local branching may occur where the network is discontinuous or misaligned. (**3**) Crack coalescence produces a continuous fracture path and releases the wafer. The measured separation stress therefore reflects the residual resistance of the prepared crack network, as well as the test configuration [3,11,27].

Direct mechanical separation is both a processing route and a diagnostic of the modified structure (Figure 3). Bonded fixtures commonly apply tension normal to the intended release plane; however, fixture and specimen compliance can introduce mixed-mode loading. The measured force–displacement curve can reveal progressive damage, abrupt instability or incomplete release. Fractography is needed to establish whether the peak corresponds to rupture within the modified plane, edge fracture or adhesive debonding [3,6,11].

For direct mechanical lift-off, tension normal to the modified plane gives the cleanest measure of residual bonding because it primarily supplies mode-I opening. Peel and shear can reduce fixture requirements but add mode II/III components and may encourage out-of-plane branching. KABRA-type manufacturing follows the same general sequence of buried laser modification, followed by applied-force separation, with the practical load strongly dependent on how completely the laser step has linked the crack network [8,21].

Within-study comparisons demonstrate the sensitivity to the initial crack state. For n-type specimens irradiated from the Si-face, Yao et al. reported average separation stresses of 3.20 and 2.12 MPa for scanning along $\left[1\;1\;\bar{2}\;0\right]$ and $\left[1\;\bar{1}\;0\;0\right]$, respectively [11]. This represents a calculated reduction of 33.8% under a matched change in orientation. Youn et al. obtained separation below 5 MPa in favorable configurations, whereas several configurations did not separate within the 38 MPa instrument limit [1]. Wang et al. reported an optimized value of 0.04 MPa, but they also documented adhesive failure and capped loads in parts of their parameter study [3]. The lowest reported endpoint therefore has meaning only within its specimen and measurement context. Tian et al. reported that phase-corrected specimens required approximately 70% of the separation stress of uncorrected counterparts; the minimum was 1.17 MPa at 13 µJ and 25 µm scan spacing [27]. This is evidence that optical correction can improve the mechanically relevant crack network before any separation assistance is applied.

**Figure 4:**
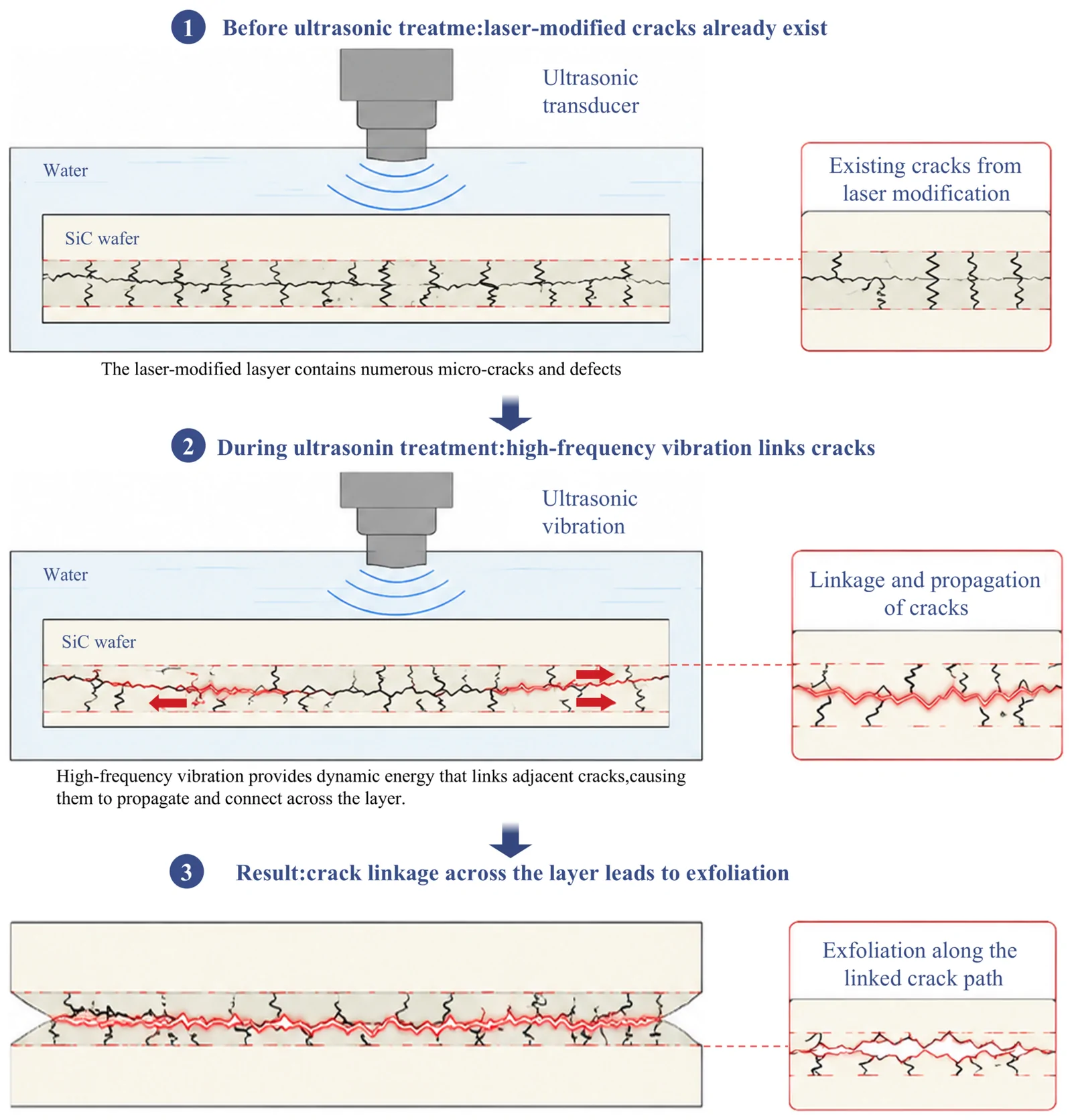
Ultrasonic-assisted separation of a laser-modified SiC wafer in an immersion configuration. (**1**) The buried modified layer initially contains cracks, defects and intact bridges. (**2**) High-frequency vibration applies cyclic loading, while liquid penetration, acoustic pressure and cavitation-related effects promote crack extension and linkage. (**3**) Once neighboring cracks become sufficiently connected, the remaining ligaments fail, and exfoliation proceeds along the laser-defined layer. The response depends on the initial crack state, ultrasonic conditions, horn-to-sample distance and liquid medium [2,8,14,38].

Ultrasonic assistance can extend the prepared cracks before or during mechanical release (Figure 4). Directly coupled vibration produces cyclic stresses, while immersion adds acoustic pressure, fluid motion and possible cavitation near liquid-accessible regions. Wang et al. demonstrated chip separation using a Langevin transducer after laser modification; their model, informed by micro-cantilever fracture tests, connected transducer excitation to crack growth and distinguished immediate separation from a fatigue-like response at lower excitation [38]. This directly coupled approach illustrates why cavitation should not be treated as the universal explanation for ultrasonic assistance.

Modification design can also determine whether ultrasound completes release. In the off-axis study of Wang et al., an SLM split the beam along the scan direction so successive beams re-irradiated each track [16]. At a fixed 200 µm track pitch, the reported separation stresses were 0.07 MPa for three beams and 0.04 MPa for five beams, with constant power per beam and increasing total power. Specimens processed with at least three beams could be released in water using 180 W, 50 kHz ultrasound in approximately 10 s. This result concerns 10 mm square specimens mounted on coverslips. It demonstrates coupling between repeated modification and acoustic release, rather than a full-wafer throughput benchmark.

For immersion treatment, Lv et al. [8] used an orthogonal design varying frequency, duration and power. Their reported baseline stripping force was 30 N, and the lowest force in the design was 12.167 N after treatment at 20 kHz and 700 W for 5 min, giving a calculated reduction of 59.4%. Because several factors changed between the baseline and treatment, the result describes the combined treatment rather than an isolated power effect. In a subsequent batch, adjusting the tool–sample gap enabled release during ultrasonic exposure and supported a 6-inch demonstration. The reported areal roughness was approximately 5.61 µm without ultrasound and 5.98 µm with assistance: reduced separation resistance did not produce a corresponding improvement in surface roughness.

Immersion introduces additional mechanisms beyond cyclic solid vibration. Cavitation bubbles can nucleate near liquid-accessible crack mouths; collapse then produces short pressure pulses and microjets that act on the crack faces. Lv et al. estimated jet velocities of several hundred meters per second in their mechanistic analysis [8], while later fluid-mediated work predicted strong pressure amplification near crack tips [2]. These estimates support a cavitation contribution but should be interpreted together with crack accessibility and acoustic coupling, since directly coupled ultrasonic separation can also occur without an immersion cavitation mechanism [38].

He et al. extended the investigation to glycerol concentration, temperature and tool distance [2]. Their analysis identified glycerol fraction and tool distance as significant factors, whereas temperature was not significant over the tested 20–40 °C range at the stated 95% confidence level. Pure water at 40 °C and a 1 mm distance nevertheless gave the lowest measured force, 10.5 N, in a confirmation test. More generally, medium-dependent force reduction supports a role for fluid dynamics but does not independently establish the pressure acting at buried crack tips. Bubble-collapse simulations require validation against the accessibility, confinement and acoustic conditions of the actual crack network.

The practical ultrasonic window is bounded by incomplete linkage at insufficient excitation, and fragmentation at excessive exposure or unfavorable tool spacing [8,14]. Treatment time, residual force, edge integrity and the fraction of intact released wafers must therefore be assessed together. A low post-treatment force records reduced residual resistance; spontaneous release during exposure records completion of the separation operation. Neither measurement, by itself, establishes a low complete-cycle cost.

### 3.2. Cold Splitting

**Figure 5:**
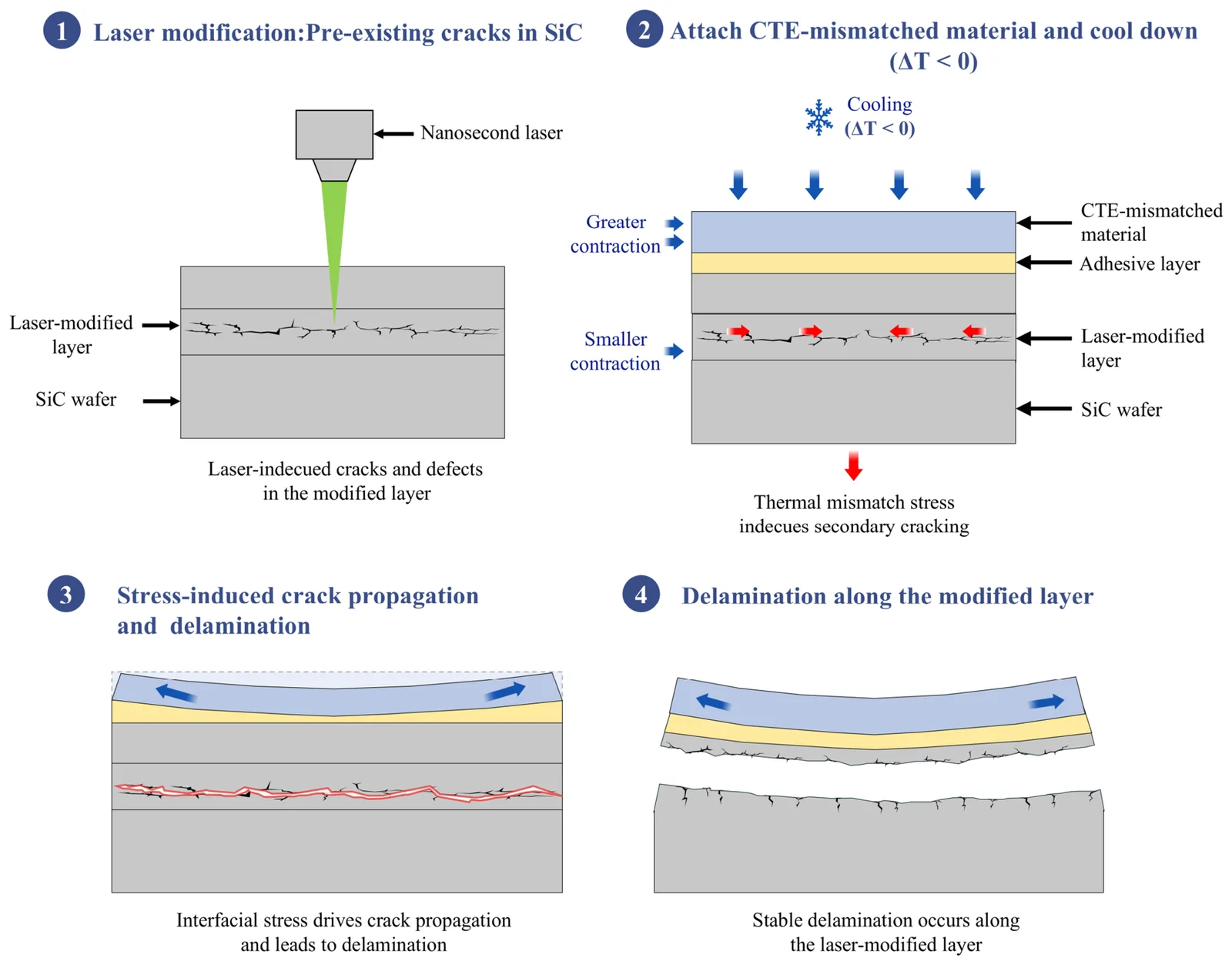
Polymer- or stressor-assisted cold splitting after laser modification. (**1**) Laser processing first creates a buried weakened plane. (**2**) A bonded layer with a different coefficient of thermal expansion is attached, and the assembly is cooled; in COLD SPLIT, cooling also markedly increases polymer stiffness. (**3**) Differential contraction generates bending and interfacial stresses that drive crack propagation toward and along the conditioned plane. (**4**) Stable delamination then releases the wafer.

Cold splitting generates separation-driving stresses through the differential contraction of a bonded polymer and SiC (Figure 5). Cooling changes both mismatch strain and polymer stiffness, and the resulting load is transmitted through the bonded assembly to the crystal. Kaule et al. emphasized the increase in polymer stiffness on cooling through its glass transition and the requirement for relative contraction of the polymer [9]. Thus, low temperature is not itself the fracture mechanism: useful loading requires a suitable combination of thermal contraction, stiffness, adhesion and geometry.

In the reported COLD SPLIT sequence, surface conditioning and internal laser writing are followed by a primer, a sacrificial layer and a millimeter-scale polydimethylsiloxane stressor. Cooling through the polymer glass-transition regime raises its stiffness while differential thermal contraction stores the energy needed for crack propagation; liquid-nitrogen cooling can then complete splitting on a time scale of tens of seconds [9,10]. The sacrificial layer enables removal of the polymer after release. This architecture also provides temporary support to the thin wafer, which is advantageous when handling, rather than fracture resistance, becomes the limiting factor.

Laser conditioning supplies a preferred path for the spalling crack. Swoboda et al. reported laser-assisted SiC cold splitting with as-spalled arithmetic roughness below 4 µm and described processing of SiC substrates up to 4 inches [10]. Their process includes surface preparation, internal modification, sacrificial-layer application, polymer lamination, cooling, release and polymer removal. This sequence makes clear that the nominal two-step route contains auxiliary operations essential to practical wafer recovery. Kaule et al. subsequently demonstrated the broader control of crack-growth features using laser conditioning; the 300 mm substrate demonstration and associated submicrometer roughness in that work concerned silicon and should remain distinct from the SiC evidence [9].

Fracture-path selection depends on the competition between the stress-layer geometry and the weakened internal region. The classical film–substrate treatment of Suo and Hutchinson explains why stressor thickness and elastic mismatch influence the steady propagation depth [17]. Recent controlled spalling of 4H-SiC using nickel stressors further shows the importance of stressor uniformity and reliable edge initiation [39]. That study did not use a buried laser-modified layer and is therefore a mechanistic comparator rather than a demonstration of the same two-step process. Together, these studies indicate that laser conditioning guides a mechanically driven crack but does not eliminate the need to control initiation and loading over the full area.

The polymer can support the separated wafer, which is potentially useful as wafer thickness decreases. The benefit must be weighed against debonding, cleaning and resurfacing requirements. A favorable crack path can reduce finishing, whereas local departure from the conditioned region can leave steps or deep defects on either newly exposed face. Cold splitting should therefore be assessed through repeated wafer recovery and ingot reuse, with the full bonding–cooling–release sequence included in throughput calculations.

Laser conditioning markedly improves path control compared with uncontrolled spalling. Reported SiC demonstrations include wafers below 200 μm and a minimum thickness of about 74 μm, together with as-split roughness of roughly 2 μm in 4H-SiC [9,10]. The often-cited 300 mm demonstration and submicrometer roughness reported in the same development program concern silicon and should not be treated as direct 300 mm SiC evidence [9].

### 3.3. CW Laser and Other Thermal Stress-Driven Splitting

**Figure 6:**
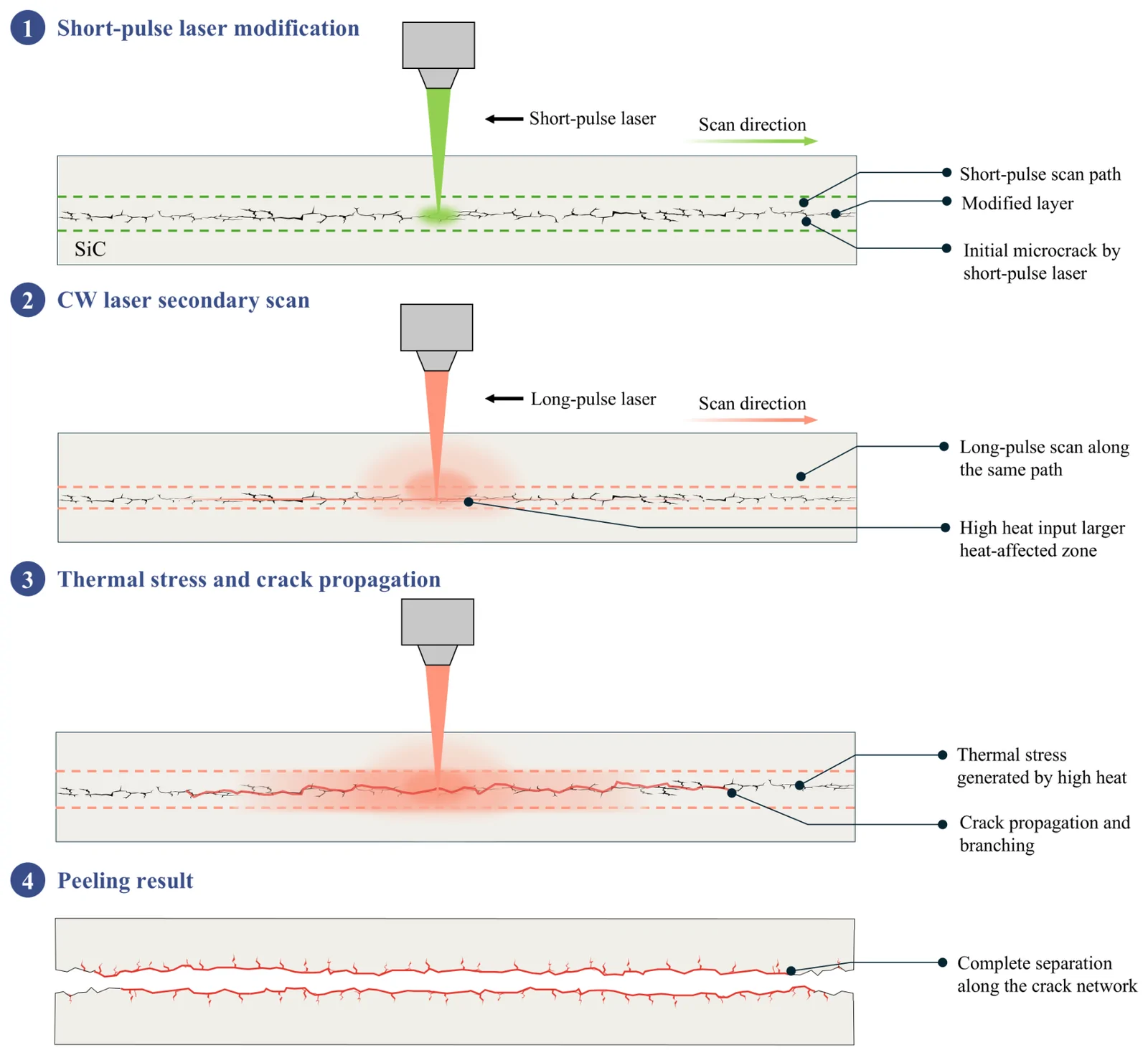
Continuous-wave (CW) laser-assisted splitting after short-pulse subsurface modification. (**1**) A short-pulse laser first creates a crack-containing modified layer with enhanced optical absorption. (**2**) A secondary CW beam is scanned along the prepared path and preferentially heats this layer. (**3**) Local temperature gradients and constrained thermal expansion generate stresses that extend and connect the cracks. (**4**) Once a continuous network is established, wafer release can occur with little or no external mechanical force. The usable process window is bounded by insufficient thermal driving and excessive heating or edge damage [15].

CW-laser splitting uses a second optical treatment to load the region prepared by a pulsed laser (Figure 6). Jiang et al. showed that picosecond modification increased absorption at 1064 nm, allowing subsequent CW irradiation to concentrate heating around silicon- and carbon-containing modified material [15]. The initial treatment therefore supplies both optical contrast and a mechanically susceptible region. Local thermal expansion, temperature gradients and further transformation of the modified material then promote separation.

Their controlled experiments varied CW power and scan spacing while retaining the same initial picosecond treatment. At a spacing of 0.02 mm and 35 W, the residual splitting tensile strength fell to approximately 4 MPa. Increasing power under tightly spaced scanning could damage the specimen edge, whereas 105 W at a spacing of 0.04 mm enabled direct splitting of 10 × 10 × 0.5 mm specimens without externally applied tensile loading [15]. This is evidence for a processing window, not a monotonic benefit from increasing heat input.

The separated surfaces also retained evidence of the thermal history. Jiang et al. identified compressive residual stress and silicon- and carbon-containing surface material with a reported thickness of approximately 50–250 nm [15]. These findings distinguish optical release from a finished substrate. A thin transformed surface layer does not independently bound the depth of fracture-related damage or the finishing allowance. Thermal models likewise need to resolve where opening and closing stresses occur: a large temperature rise alone does not establish favorable loading of a particular crack.

All-laser separation extends beyond CW irradiation. Liu and Hong used an initial high-fluence pulsed treatment to generate internal microcracks and enhance absorption, followed by lower-fluence pulses to promote their extension and interconnection [40]. Their subsequent heat-assisted approach maintained approximately 20 × 20 mm specimens at 400 °C during pulsed processing, reducing the temperature contrast around the irradiated region; an areal roughness of 89.3 nm was reported under optimized conditions [41]. The latter integrates thermal assistance with modification and growth rather than adding a distinct post-modification cooling step. It nevertheless demonstrates that controlling the spatial uniformity of thermal loading can be as important as increasing its magnitude.

Thermal coupling can also be moved into the writing step itself. Xie et al. used synchronous dual-beam nanosecond irradiation so that adjacent foci shared a transient heated region; at 60 μm spacing the reported peeling stress decreased from 6.281 to 4.116 MPa, whereas the benefit was much smaller at 100 μm spacing [37]. In the sub-nanosecond regime, repeated absorption by laser-generated products can similarly promote cracks that propagate ahead of later scan lines, reducing the final nominal separation stress to about 0.04 MPa under optimized conditions [3]. Both results show that the most effective thermal assistance may be to increase crack connectivity before the nominal separation step begins.

Thermal-stress splitting is, accordingly, an umbrella mechanism that includes cold splitting, localized optical heating and combined thermal treatments. Uniform heating of an unconstrained homogeneous crystal does not automatically generate a useful separation stress; temperature gradients, expansion mismatch or other constraints are required. Geometry must also remain explicit. The pulsed/CW method of Zhang et al. and the layered stealth-dicing method of Yang et al. concern die singulation through wafer thickness [20,42]. Song et al. likewise used sequential nanosecond exposures to extend cracks toward the surface and reduce edge defects in stealth dicing [43]. These studies support control of crack initiation and growth through staged irradiation, but cut-edge quality and three-point-bending measurements cannot be transferred directly to horizontal wafer release.

### 3.4. Chemical and Hybrid Separation

Chemical separation exploits changes in the reactivity or electronic structure of the modified region. It can reduce the amount of solid material that must be fractured, provided that the reagent reaches the buried layer and reacts selectively. Geng et al. combined femtosecond modification with bandgap-selective photoelectrochemical removal [7]. The modified region contained amorphous silicon and carbon and had a reported optical bandgap near 0.4 eV. Visible illumination and electrical bias in a hydrofluoric acid (HF)-containing electrolyte promoted selective removal, producing release of 10 × 10 mm specimens after 180 min with arithmetic roughness of approximately 1 µm.

The photoelectrochemical route is especially selective because the laser-damaged layer has a much smaller effective optical gap than pristine 4H-SiC. Under visible illumination and bias, the modified material can be oxidized and dissolved preferentially while the surrounding crystal remains largely unaffected [7]. Geng et al. [7] released 10 × 10 mm specimens after about 180 min and reported roughness near 1 μm, but the rate was limited by transport through the narrow buried gap. Chemical selectivity can therefore minimize mechanically induced branching, while throughput remains controlled by reagent access and product removal.

This route illustrates both the advantage and the limitation of selective removal. The wafer can be released without forcing cracks through every remaining ligament, but the process depends on transport along a confined internal region. Geng et al. observed etching from accessible edges and identified mass transfer as a rate limitation [7]. As the release area increases, reagent supply and reaction-product removal become coupled to the evolving gap. The processing time of a small specimen therefore provides no simple linear prediction for a full wafer.

Li et al. combined ultrasonic treatment with KOH solution and an improved laser pattern [14]. For the original modification, the reported force decreased from 79 N without assistance to 33.74 N after ultrasonic treatment in deionized water and to 26.85 N in KOH solution. The change from water to KOH corresponds to a calculated additional reduction of 20.4%; the 66.0% reduction relative to 79 N includes both ultrasonic and medium effects. Their dual-modification strategy first introduced low-energy auxiliary tracks and then higher-energy crack-inducing tracks between them, controlling where staggered cracks connected. This reduced the force further to 19.85 N, or 26.1% below the original-pattern KOH result. These comparisons identify separate opportunities in liquid-mediated treatment and initial crack-network design.

The coupon values must remain distinct from the same study’s 6-inch demonstration. The observed acceleration of liquid renewal and reaction-bubble generation under ultrasound supports coupled transport and chemical action. Quantifying a super-additive interaction would additionally require matched force measurements for chemical-only, ultrasonic-only and combined treatments. The strongest case for a hybrid route is a reproducible improvement in finished-wafer yield or material utilization that justifies its additional operations.

## 4. Performance Comparison and Challenges

### 4.1. Comparing Separation Performance and Surface Integrity

**Figure 7:**
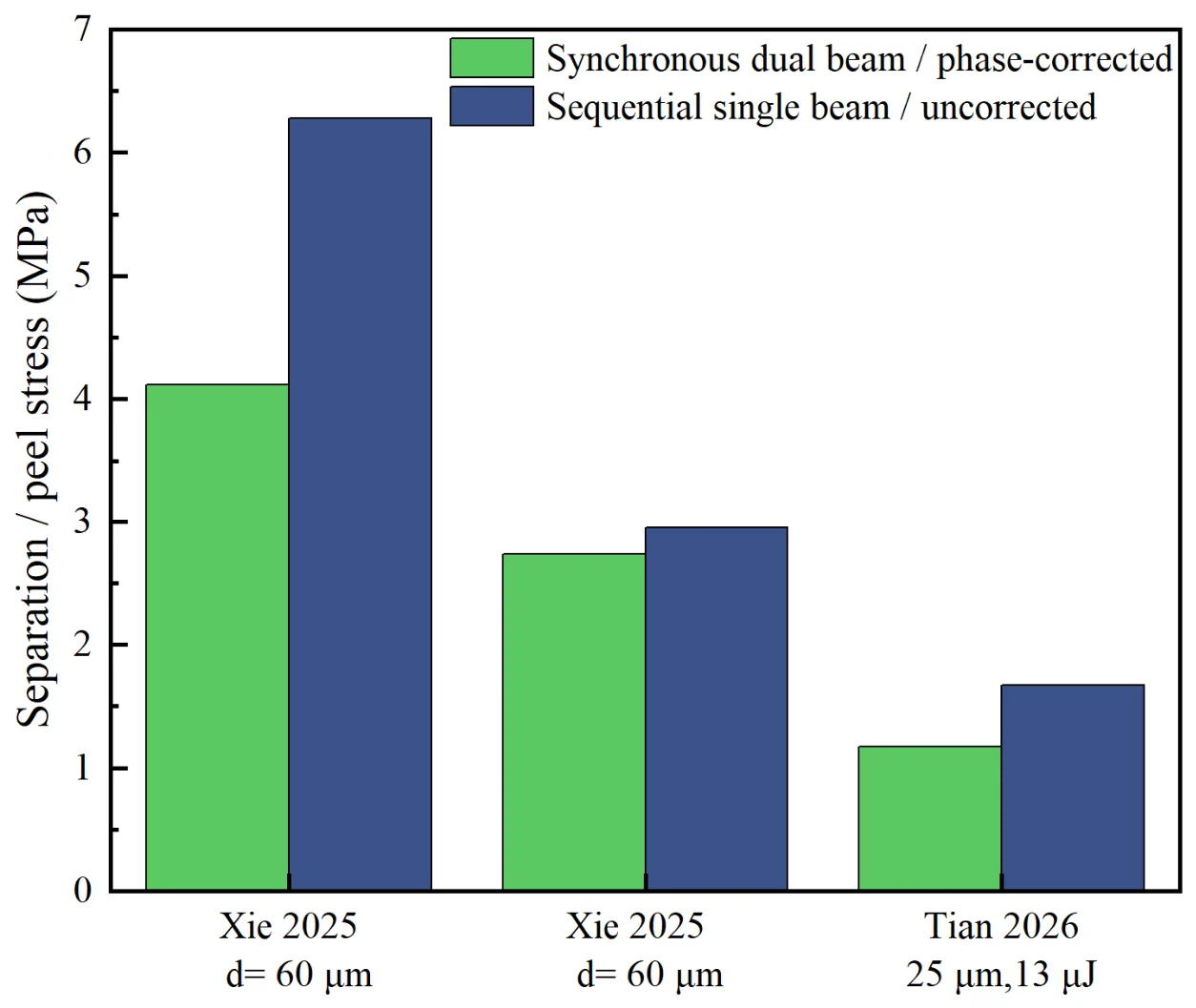
Matched reductions in separation or peel stress produced by synchronous dual-beam writing and phase-corrected picosecond processing. Xie et al. [37] compare synchronous and sequential writing at line spacings of 60 and 100 μm; Tian et al. [27] compare phase-corrected and uncorrected processing at 25 μm spacing and 13 μJ.

**Figure 8:**
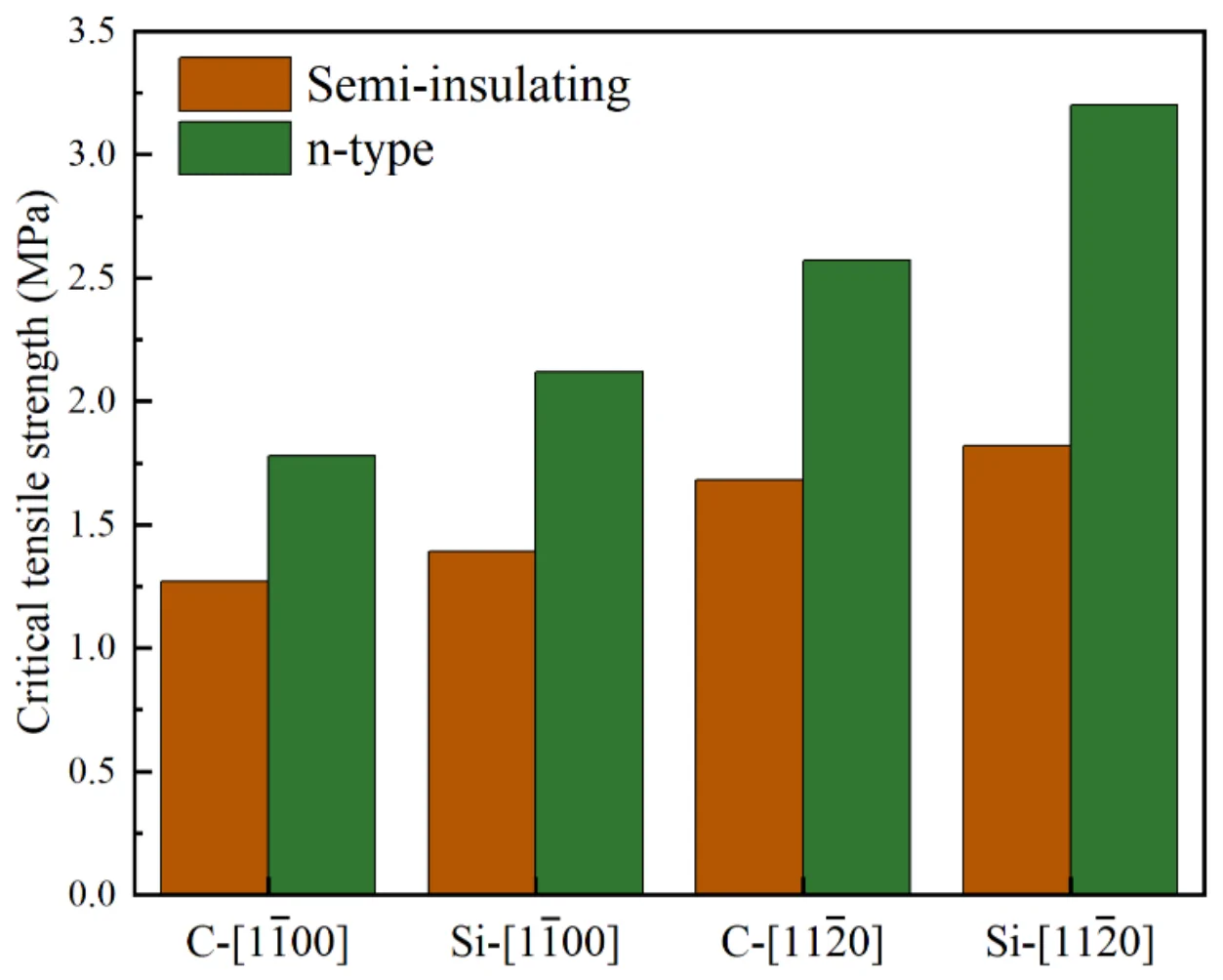
Critical tensile strength after picosecond modification as a function of incident face, scan direction and doping. The data show consistently higher resistance for n-type than semi-insulating 4H-SiC and the lowest values for C-face incidence with the favorable in-plane scan direction [11].

**Figure 9:**
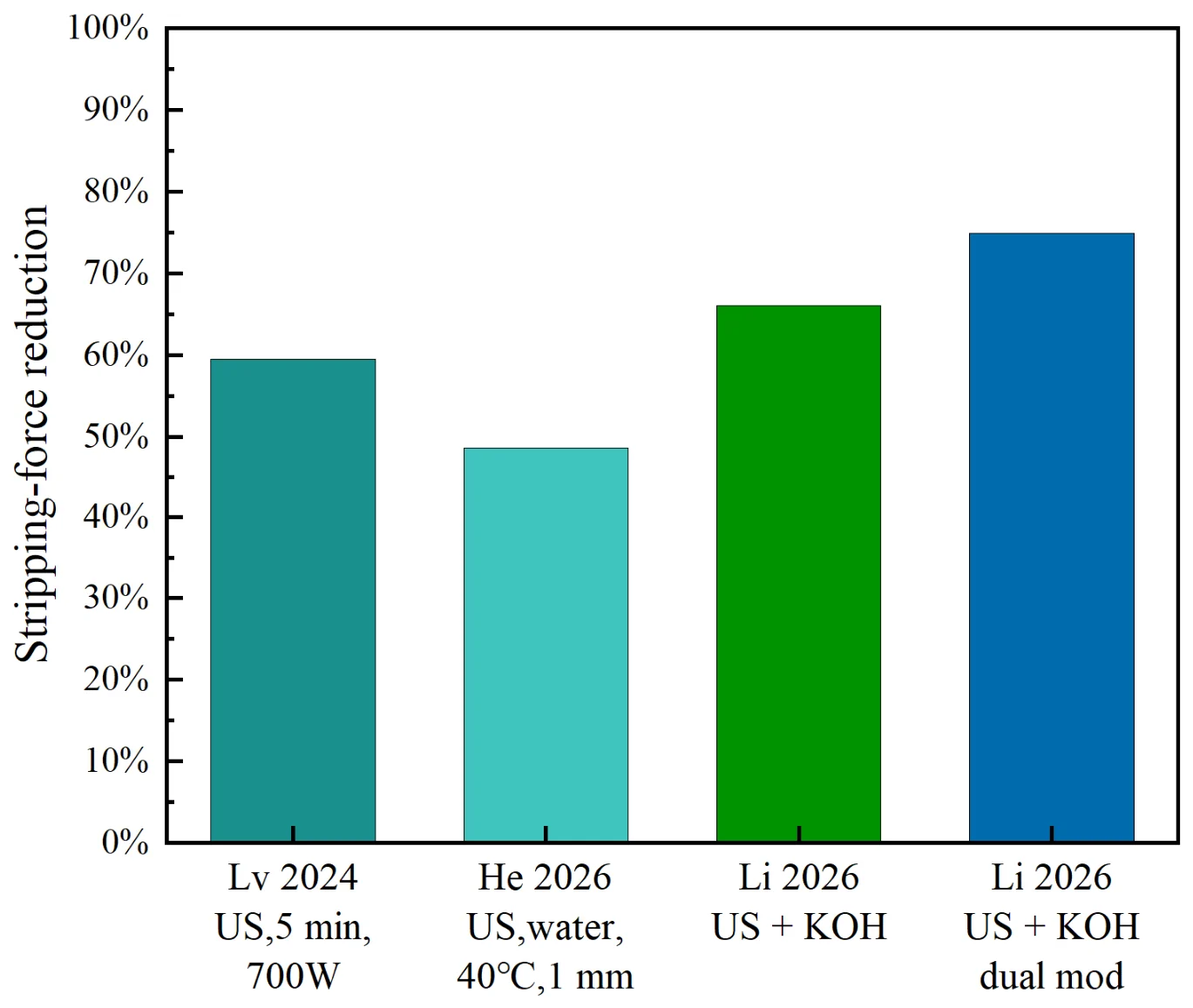
Reported reduction in stripping force produced by ultrasonic and hybrid assistance. Lv et al. [8] report the optimized ultrasonic treatment relative to their baseline; He et al. [2] report a 48.6% reduction in the abstract; however, the baseline value tabulated in that paper is numerically inconsistent with this percentage; Li et al. [14] show the additional effects of KOH medium and dual-modification crack linking.

**Figure 10:**
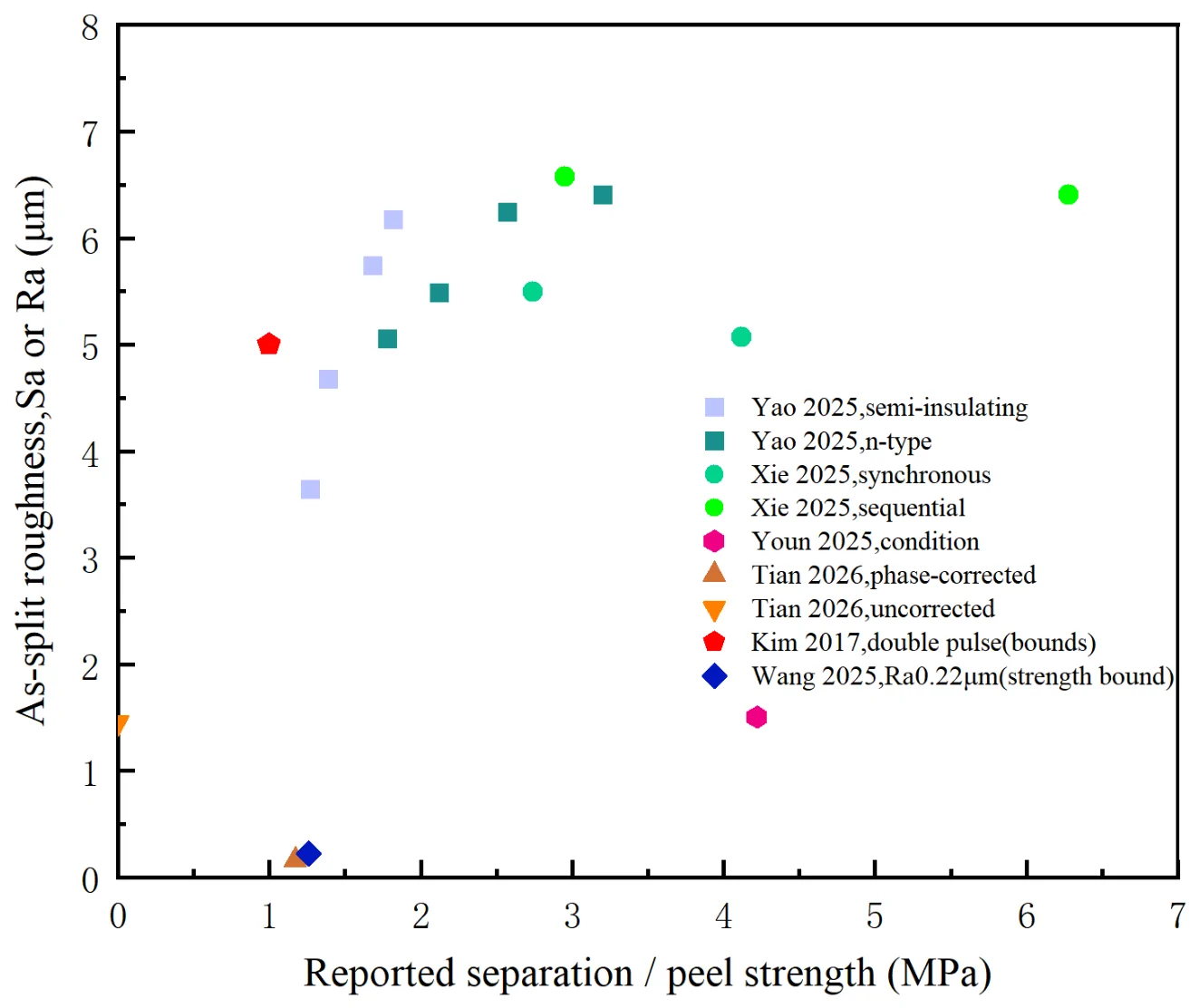
Reported separation or peel strength versus as-split roughness for studies that provide both metrics. Points from different studies use different roughness definitions and test configurations, so the plot is intended to show trends and bounds rather than a universal process ranking [1,3,6,11,27,37].

**Figure 11:**
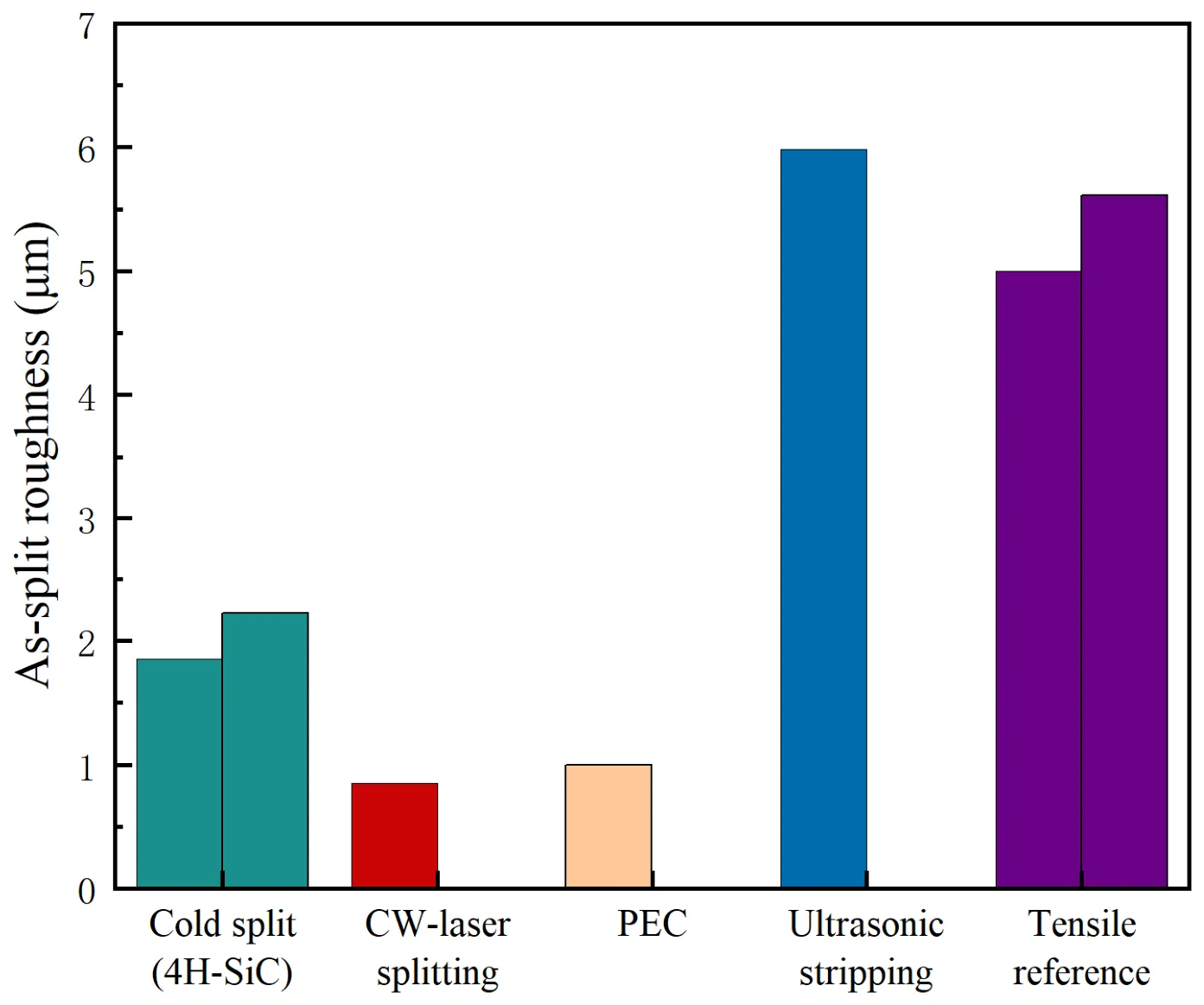
Representative as-split roughness for separation routes without directly comparable load metrics. Cold splitting, CW-laser splitting and photoelectrochemical release can produce roughness near the 1–2 μm range, whereas some ultrasonic and tensile reference conditions are substantially rougher [7,8,9,10,15].

The most defensible quantitative comparisons are those made within the same study while retaining specimen geometry, test definition and most processing conditions. Table 1 summarizes representative laser conditions and reported separation outcomes, while Figure 7, Figure 8, Figure 9, Figure 10 and Figure 11 compile the controlled comparisons discussed below. Matched changes in orientation, beam timing or medium are distinguished from comparisons between an untreated baseline and a treatment selected through a multifactor design. These results establish controllability of separation resistance; they do not constitute a ranking of manufacturing routes.

The headline ranges quoted in this review, approximately 0.04 to >38 MPa for reported separation strength and approximately 0.16 to 6.5 μm for commonly reported as-split roughness, should therefore be interpreted as envelopes of the published literature, not as a pooled dataset. Direct numerical comparison is defensible only when loading mode, loaded area, specimen dimensions, crystal face and orientation, off-cut, failure location, roughness definition, sampling area, and instrument protocol are matched. Across unmatched studies, the ranges demonstrate process sensitivity but should not be used to rank separation routes quantitatively.

Several measurement distinctions are essential (Table 2). Force in newtons and nominal stress in megapascals should not be pooled without the loaded area and fixture mechanics. An instrument-limited test, adhesive failure and complete release during ultrasonic or thermal treatment represent different outcomes. In particular, zero subsequent mechanical stripping force means that another driving field has already completed separation; it does not imply zero fracture resistance. Likewise, predicted acoustic pressure or thermal stress is not an alternative experimental measurement of separation strength [1,3,8,15].

Surface integrity requires more than one roughness number. Line-based arithmetic roughness, areal arithmetic roughness and root-mean-square roughness use different definitions and sampling procedures. Their values should retain the measurement scale and surface condition. Kim et al.’s approximately 5 µm root-mean-square roughness, Han et al.’s approximately 1.8 µm reported roughness, and the areal values in heat-assisted processing describe different experiments [6,13,41]. A direct phase-correction comparison by Tian et al. is more informative than a cross-study ranking: the reported areal roughness, Sa, decreased from 1.45 to 0.16 µm, while maximum height, Sz, decreased from 12.09 to 3.33 µm [27]. These results apply to the compared on-axis specimens and measured surface areas. By contrast, Li et al. reported a Sa of 19.51 µm after their combined dual-modification and ultrasonic–KOH treatment, despite its low residual force [14]. None of these values alone determines the removal needed to obtain an acceptable substrate. Isolated deep steps, subsurface cracks, residual phases, bow and total thickness variation can control finishing even when average roughness is low.

Adjacent cutting studies clarify additional sources of damage. Xu et al. varied femtosecond peak power and scan speed and characterized cut-mark width and groove depth [44]; these measures do not establish a buried release plane or finished-wafer yield. In 355 nm surface cutting of 6H-SiC, Tseng et al. found stronger oxidation with increased energy input or repeated exposure [45]. Hybrid dicing of metallized 4H-SiC introduces further interactions: Li et al. linked longer grinding-induced coupling cracks to carbon-particle formation and increased sidewall roughness [46], while Qu et al. distinguished alloy tearing during stealth dicing from thermally induced interfacial peeling during ablation-assisted dicing [47]. These results show why crack access, surface chemistry and adjoining layers need separate characterization; they are not additional horizontal-wafer separation benchmarks.

Figure 7, Figure 8, Figure 9, Figure 10 and Figure 11 consolidate the quantitative comparisons already discussed while keeping unlike metrics separate. Figure 7 shows matched reductions in separation or peel stress produced by beam-timing and phase-correction changes [27,37]. Figure 8 isolates the combined effects of incident face, scan direction and doping [11]. Figure 9 summarizes reported ultrasonic and hybrid stripping-force reductions [2,8,14]. Figure 10 compares strength with as-split roughness where both quantities are available, and Figure 11 collects roughness values for routes whose separation loads are not directly comparable. Together they support a central conclusion of the review: lower separation resistance does not automatically imply a smoother released surface.

### 4.2. Material Utilization and Manufacturing Efficiency

Kerf reduction is an important benefit of fracture-assisted wafering, but total material consumption includes more than the directly modified or removed layer. A complete accounting includes wafer-side finishing, resurfacing of the remaining ingot, cropping and losses through breakage or rejection. The modified-layer thickness, fracture opening and total thickness consumed per finished wafer are different quantities and should be reported separately. This distinction prevents a narrow optical track from being interpreted as an equally small manufacturing loss [3,10]. For example, Wang et al. reported a directly modified layer below 5 µm but an overall post-separation damage thickness below 30 µm in their off-axis multi-beam study [16]; these describe different spatial measures. Suppressing secondary lower modification, as demonstrated by Tian et al., addresses another component of the finishing allowance that surface roughness alone can miss [27].

Repeated use of the remaining crystal is the appropriate test of material recovery. A method can produce an acceptable wafer while leaving deep damage on the ingot, increasing the loss in the next cycle. Conversely, improving the consistency of the fracture depth can reduce the finishing burden on both faces. Controlled spalling experiments outside the laser-modified route demonstrate how reconditioning and repeated release can be evaluated explicitly [39]; equivalent repeated-cycle evidence is needed to substantiate yield gains for laser-assisted wafering.

Throughput needs the same consistent boundary. Scanning time, ultrasonic exposure, cooling time or chemical release time is only part of the production cycle. Bonding, alignment, transfer, debonding, cleaning, finishing and inspection can change the relative advantage of each route. Six-inch demonstrations establish important progress in modification and release [3,8,11,14,30], but manufacturing readiness additionally requires wafer-area uniformity, reproducibility across ingots and statistical finished-wafer yield. Scaling changes the load distribution, heat flow, acoustic field and transport distance; it is not simply an increase in the scanned area.

### 4.3. Joint Process Design and Predictive Control

The optimal first-stage condition depends on the intended second stage. Mechanical release favors connected, nearly coplanar cracks with limited remaining ligaments. CW irradiation additionally requires useful absorption contrast and controlled heat deposition. Fluid-mediated methods require access to the crack network, while chemical methods require sufficient selectivity between modified material and intact SiC. These requirements can conflict: more extensive modification may improve absorption or chemical access while increasing the material that must be removed later [7,14,15]. Process optimization should therefore seek reliable release with limited finishing rather than the minimum separation load alone. The recent reviews likewise identify beam shaping, hybrid processing and feedback control as development priorities [5,21,23]. The newer primary evidence makes this requirement concrete: phase correction controls unwanted depthwise modification, while repeated multi-beam irradiation changes both track resistance and lateral crack connectivity [16,27].

In practice, joint optimization can be implemented as a closed-loop sequence rather than as independent parameter sweeps. First, laser wavelength, pulse duration, pulse energy, repetition rate, numerical aperture, focal depth, scan direction and line interval are selected to target a specified modified-layer depth, crack reach and ligament fraction. Second, the prepared plane is mapped before separation using optical/OCT or other non-destructive imaging, supplemented by local Raman or cross-sectional microscopy. Third, the least severe separation treatment capable of completing release is selected (normal tension, ultrasound, thermal mismatch, CW heating or chemical assistance). Finally, both newly exposed surfaces are measured for step height, roughness and subsurface damage, and the finishing allowance is fed back to the next laser/separation cycle. The optimization objective should therefore minimize a combined cost of release failure, damage depth, finishing removal and cycle time rather than separation load alone.

Predictive modeling can help resolve these trade-offs when its inputs describe the observed structure. Coupled laser-interaction models connect energy deposition to stress generation [29], and phase-field fracture formulations provide a basis for simulating crack initiation, turning and coalescence without prescribing every subsequent crack path [48]. Atomistic simulations by Han et al. connect thermal loading, structural transformation and dislocation-mediated crack deflection, but they use a cubic SiC model whose crystallographic paths cannot be transferred directly to 4H-SiC [49]. Yu et al. linked atomistic traction–separation behavior to cohesive-zone simulations of polycrystalline SiC [50]; their grain-dependent model provides a multiscale methodology rather than validation for laser-modified single-crystal wafering. Application to that problem requires orientation-dependent fracture resistance, realistic initial cracks and suitable loading boundaries. A model calibrated only against peak force may reproduce the experiment for the wrong geometric reason. Validation should also consider the separation trajectory, load–displacement evolution and resulting surface steps.

The immediate experimental priority is to connect measurable initial-state descriptors to the probability of obtaining an acceptable wafer. Two descriptor sets should be distinguished. Incoming-material descriptors should include polytype; Si- or C-face; off-cut angle and azimuth; doping type/concentration or resistivity; wafer/ingot thickness; bow and warp; initial surface roughness; optical transmission; and defect density (including micropipes or other extended defects, where relevant). Among these, crystal face/orientation, off-cut geometry and doping-dependent optical absorption are expected to exert the strongest systematic influence on the laser-generated crack state, while local defects contribute scatter. Pre-separation descriptors should then quantify the modified structure itself: mean modified-plane depth and its variation, modified-layer thickness, lateral crack length, connected-crack area fraction, ligament width or area fraction, vertical crack offset, residual stress, and the occurrence of edge discontinuities. Measurements before and after assistance can then distinguish inadequate initial connectivity from damage introduced during separation.

For cross-study comparison, a minimum reporting set should accompany future separation tests: specimen area and thickness; modified-plane depth; bonding and fixture configuration; loading mode and rate; peak force and area-normalized nominal stress; the number of successful and unsuccessful repeats; as-split Sa or Ra with the measurement area; maximum step height; and cross-sectional laser-damage thickness. Force in newtons should not be converted to stress unless the loaded area and lever or fixture mechanics are stated. A standardized stripping protocol of this kind would make separation resistance, surface integrity and material utilization directly comparable across laboratories [1,2,3,6,8,11].

### 4.4. Experimental Validation of Crack Connectivity and Standardized Characterization

The fracture sequence in Figure 2 should be interpreted as a mechanistic synthesis of published observations, not as direct imaging of every stage in a single specimen. Nevertheless, recent primary studies provide much of the required experimental foundation. Cross-sectional SEM has directly resolved lateral cracks and nanometer-scale crack gaps in laser-modified 4H-SiC, while spatially resolved Raman measurements have mapped the associated tension–compression residual-stress field [51]. A recent multi-technique study combined SEM/EDS, micro-Raman mapping, angle-resolved XPS, SIMS and site-specific FIB-TEM/EDS/SAED to resolve laser-sliced SiC from the micrometer to nanometer scale [52]. Raman analysis of internally modified SiC has also distinguished crystalline SiC, amorphous SiC, amorphous Si and amorphous carbon while linking scan strategy to delamination performance [53]. What remains largely missing is same-specimen, three-dimensional tracking of a defined set of crack contacts and ligaments from the modified state through final rupture.

A practical distinction between crack linkage through an intact ligament and linkage through previously modified material requires correlative morphology and phase identification. Intact 4H-SiC should retain a strong crystalline Raman signature and crystalline diffraction, whereas modified bridges may show attenuation or broadening of the 4H-SiC modes together with amorphous-Si, amorphous/graphitic-C or 3C-SiC signatures. After separation, paired locations on the released wafer and remaining ingot should be registered to the pre-separation map. Fresh crystalline cleavage across a bridge, with little transformed material, supports ligament rupture; persistent transformed phases, recast material or void-rich zones on both matching surfaces support failure through the modified region. EDS or XPS can provide complementary chemistry, while TEM/SAED can determine local phase and orientation.

The required spatial resolution is hierarchical. Full-area optical, infrared or OCT mapping is appropriate for micrometer-scale depth nonuniformity and incomplete regions; SEM and FIB-SEM are required for sub-micrometer ligament morphology and tens-of-nanometers crack openings; and FIB-TEM with diffraction/EDS resolves local phase, misorientation and nanoscale damage. Micro-Raman mapping provides approximately micrometer-scale phase/stress information and is therefore well suited to crack-tip and ligament maps. EBSD, preferably on a carefully polished cross-section, can quantify crystal orientation and local misorientation across wider ligaments, while TEM diffraction is preferable for the narrowest bridges. Cathodoluminescence can complement these methods by mapping defect-related electronic contrast, but it should not replace direct structural imaging. The broader fracture-toughness literature further shows that the measured fracture resistance of SiC depends strongly on test method, scale, temperature and crystallographic anisotropy; these dependencies should therefore be considered when interpreting local ligament failure and selecting mechanical descriptors for laser-guided fracture [54].

To correlate connectivity with separation stress on the same specimen, the crack network should first be mapped and reduced to quantitative descriptors such as connected-crack fraction, residual ligament fraction, lateral crack length-to-pitch ratio and the distribution of vertical crack offsets. The specimen should then be loaded in a defined normal-tension geometry while recording the complete force–displacement curve and failure location. Registration of the post-fracture surfaces to the pre-test map would allow the measured nominal separation stress to be related directly to the initial connectivity metrics rather than to laser settings alone.

### 4.5. Cross-Laboratory Normalization, Production Readiness and 3–5 Year Priorities

Cross-laboratory comparison should combine complete raw laser parameters with a small set of derived metrics (Table 3). Useful process-normalization quantities include line energy, Pavg/v; areal scan energy, Pavg/(v d); normalized modification depth, h/t; normalized damage thickness, td/t; and a crack-interaction ratio, such as d/(2Lc), where d is line pitch and Lc is the mean lateral crack length from one track. The residual ligament or connected-crack area fraction should be reported whenever it can be measured. These descriptors help compare geometries, but they cannot replace wavelength, pulse duration, numerical aperture, polarization and focal conditions because nonlinear absorption and aberration make equal nominal energy densities physically non-equivalent.

For a round-robin reference test, we recommend a common coupon geometry (for example, a 10 × 10 mm specimen with the modified plane near mid-thickness), normal loading to a documented bonded area, a fixed loading rate and at least five replicates per condition. Force in newtons should be retained, together with the area-normalized nominal stress, and peel, shear or lever-based tests should not be collapsed into the same metric. A shared reference 4H-SiC material lot, face/off-cut specification and baseline surface condition would allow laboratories using different laser platforms to distinguish equipment effects from material variability.

Production readiness requires evidence beyond a single successful low-load release. At the intended commercial diameter—ultimately 200 mm for current scale-up targets—a convincing pilot study should report the distribution of released-wafer thickness and total thickness variation, bow/warp, edge-chip or edge-crack statistics, post-finish roughness and subsurface damage, material removed from both the wafer and the remaining ingot, complete cycle time, and intact-wafer yield. As a proposed statistical benchmark rather than an established industry standard, at least 30 consecutive release cycles drawn from multiple ingots would provide a much more meaningful process-capability test than isolated best-case wafers. Downstream thickness and surface acceptance limits should be tied explicitly to the intended device-manufacturing specification. For thermally assisted routes, it is also important to distinguish moderate process heating from severe thermochemical exposure. Studies of HfB2-SiC ultra-high-temperature ceramics in high-enthalpy dissociated-air flows demonstrate substantial surface thermochemical evolution under extreme thermal/oxidizing conditions [55]. Although that composite system and environment differ fundamentally from single-crystal 4H-SiC wafering, the result provides a useful boundary-case reminder that phase and surface-chemistry changes should be monitored if thermal separation processes approach extreme temperatures.

For the next 3–5 years, the highest-priority activities are as follows: (1) an interlaboratory round-robin normal-tension test with common reporting fields; (2) non-destructive full-area mapping of crack connectivity and depth uniformity before separation; (3) same-specimen correlative microscopy/spectroscopy linked to force–displacement data; (4) anisotropic 3D fracture models validated against measured crack trajectories and ligament geometry; (5) closed-loop modification–separation control using in-line optical/acoustic signals; and (6) statistically documented 150–200 mm demonstrations with complete material, finishing and cycle-time budgets. Progress on these activities would convert isolated minimum-force demonstrations into transferable process windows and credible manufacturing evidence.

## 5. Conclusions and Outlook

Two-step laser-assisted SiC wafering is governed by the connection between a prepared subsurface structure and the action that completes separation. Localized modification changes composition, absorption and stress, while the geometry of the associated cracks determines the residual load-bearing network. Reliable release requires those cracks to connect along the intended depth without creating excessive damage outside it.

Mechanical tests provide a direct measure of the prepared structure’s response. Ultrasound can promote further linkage through cyclic and fluid-mediated loading; cold splitting uses contraction of a bonded stressor; and CW or pulsed optical treatments use controlled heating to extend the modified network. Chemical and hybrid approaches add selective removal and changes in fluid access. Their advantages are consequently conditional on the modification state and the required final wafer quality.

Controlled experiments support substantial improvements through orientation, scan arrangement, assistance conditions and liquid-medium selection. They do not yet justify a universal ranking based on minimum separation force or roughness. The next advance should be demonstrated through repeated production of acceptable wafers, with the separation path, complete processing time and material consumed on both sides of each split measured consistently. Designing modification and separation together offers the clearest route toward that objective.

## Figures and Tables

**Table 1:** Representative laser-modification conditions for SiC wafering.

Study	Laser	Focusing and Key Settings	Modified Layer	Separation Outcome
Kim et al. (2017) [6]	780 nm, 220 fs double pulse, 1 kHz	NA 0.8, SLM correction; ~260 µm depth; 25 µm pitch; 0.1 mm/s	Height ~15 µm; a-Si + a-C	Tensile; ≥10× lower than single pulse (~1 MPa); roughness < 5 µm; loss < 24 µm
Yang et al. (2022) [20]	1028 nm, 1–10 ps, 200 kHz, 45 µJ	NA 0.7; 20 layers; power 100 → 62%	Layered stealth-dicing tracks	Vertical dicing, 508 µm; Sa 0.7–1.6 µm
Han et al. (2022) [13]	1064 nm, 12.6 ps, 100 kHz, 30 µJ	NA 0.45; 200 mm/s; 6–10 µm interval	Damage < 2 µm	Tensile; Sa ≈ 1.8 µm; 250 µm slices
Geng et al. (2023) [7]	1030 nm, 200 fs, 58 kHz	210 µm depth; 20 µm spacing; 20 mm/s	~20 µm; gap ~0.4 eV	Tensile Ra 5 µm; PEC Ra 1 µm
Jiang et al. (2024) [15]	1064 nm, 12.6 ps + 1064 nm CW, 105 W	CW spot 100 µm; 0.04 mm spacing	~2 µm; absorption ×~2720	Split without external force; 1 cm × 1 cm × 500 µm
Lv et al. (2024) [8]	520 nm, 300 fs; 1030 nm, 15 ps; 1 kHz	80–100 µm depth	~30 µm dark band + cracks	Ultrasonic stripping; 6-inch wafer
Youn et al. (2025) [1]	1040 nm, 400 fs, 200 kHz, 5 µJ	NA 0.65; 300 µm depth; 100 mm/s; 25 µm interval	a-Si, a-C, a-SiC	4.2 to >38 MPa by scan direction; Sa 1–2 µm
Yao et al. (2025) [11]	1064 nm, 15 ps	Scan [$\bar{110}0$] or [$11\bar{20}$]; C- or Si-face incidence	Ablation lines + cracks	1.27–3.20 MPa; Sa 3.6–6.4 µm; 6-inch n-type
Wang et al. (2025) [38]	1064 nm, 1 ns, 50–150 kHz, 3–5 µJ	NA 0.65, SLM; 100 mm/s; 100 µm interval	<5 µm; graphite + a-Si	0.04 MPa minimum; Ra 0.22 µm; 6-inch
Xie et al. (2025) [26]	532 nm, 1 ns, 1 kHz, dual beam	NA 0.67; SLM beam splitting	Re-solidified + cracked zones	Peel stress 2.74 MPa; Sa ≈ 5.5 µm

**Table 2:** Qualitative comparison of the principal separation routes for laser-assisted 4H-SiC wafering. Values are representative of the cited literature and are not directly interchangeable across different test geometries.

Route	Maturity/Scale	Representative Load Response	Surface Quality	Orientation Sensitivity	Scale-Up Considerations
Direct mechanical/tensile	Most widely used laboratory benchmark; 6-inch demonstrations reported [3,11].	Nominal strength spans ~0.04 to >38 MPa because the laser-prepared crack state dominates [1,3].	Reported roughness spans sub-μm to several μm; branching can increase damage.	High: face, scan direction, off-cut and doping strongly affect resistance [1,11,16].	Simple load path, but total force and fixture/bonding uniformity become critical at 150–200 mm.
Ultrasonic-assisted	Coupon studies plus 6-inch demonstrations [8,14].	Typical reported residual-force reductions are ~25–60%; hybrid KOH/crack-linking treatments reach ~66–75% in coupon comparisons [8,14].	Often little change in roughness when the initial crack layer is unchanged.	Indirect but important through the initial crack network and access of liquid to cracks.	Horn gap, acoustic-field uniformity, immersion/drying and fragmentation risk must be controlled.
Polymer cold split	Demonstrated for SiC and mature as a stressor concept; thin-wafer release reported [9,10].	Spontaneous separation from thermal-mismatch stress; no universal force-reduction metric.	About ~2 μm reported for 4H-SiC in representative demonstrations [9,10].	Moderate to high because the laser plane must capture a mixed-mode spalling crack.	Uniform stressor thickness/adhesion, cooling, polymer removal and thin-wafer handling dominate.
CW-laser-assisted	Proof-of-concept coupon scale [15].	Direct splitting of 10 × 10 × 0.5 mm specimens without external tensile loading under optimized conditions [15].	Sub-μm to ~1 μm reported in favorable directions, but a thermally altered surface layer remains.	Primarily through the initial modified-layer absorption and crack geometry.	Heat accumulation, edge ablation and area-uniform thermal control remain unproven at full wafer scale.
In-step thermal/multi-beam	Rapidly developing; coupon and multi-beam studies [3,16,37].	Synchronous dual-beam writing reduced peel stress by 34.5% at 60 μm spacing; optimized sub-ns processing reached ~0.04 MPa [3,37].	Can improve or worsen roughness depending on overlap and crack stepping.	High because crack interaction is controlled by crystal orientation, feed direction and off-cut.	Requires stable beam shaping, focus control and uniform crack interaction over the full diameter.
Chemical/PEC/hybrid	Coupon PEC demonstrations; ultrasonic–KOH hybrid extended to 6-inch feasibility [7,14].	PEC can remove the damaged layer without a comparable mechanical separation load; ultrasonic–KOH reduces residual force substantially.	~1 μm reported for PEC; hybrid roughness depends strongly on the initial laser pattern.	Lower direct mechanical sensitivity, but crack access and modified-layer chemistry remain orientation/process dependent.	Mass transport, chemical handling, reaction time, rinsing and full-area reagent access are key limitations.

**Table 3:** Recommended correlative characterization and reporting protocol for laser-assisted SiC wafering.

Stage/Quantity	Minimum Recommended Measurement	Preferred Technique(s)	Suggested Spatial/Statistical Reporting	Purpose
Incoming crystal	Polytype; face; off-cut angle/azimuth; doping/resistivity; thickness; bow/warp; initial roughness; optical transmission; defect density.	XRD/pole figure; electrical characterization; optical/IR inspection; profilometry.	Report lot/ingot, specimen position and baseline distributions.	Separates material variability from process variability.
Modified-plane geometry before release	Mean depth and variation; layer thickness; lateral crack length; line pitch; connected-crack fraction; ligament width/fraction; vertical offsets; edge discontinuities.	Optical/IR/OCT for area mapping; cross-sectional SEM/FIB-SEM for local geometry.	Full-field map plus site-specific sections; report mean, SD and extrema.	Quantifies the actual fracture network rather than nominal laser settings.
Phase and chemistry at crack contacts/ligaments	4H-SiC crystallinity; a-Si/a-C/graphitic-C/3C-SiC signatures; Si:C/O composition.	Micro-Raman mapping; EDS; XPS/ARXPS; FIB-TEM/SAED/EDS; SIMS when depth chemistry is needed.	Register maps to crack tips, ligaments and modified tracks; include pristine reference.	Distinguishes rupture of intact ligaments from failure through transformed material.
Residual stress/local orientation	Raman peak shift; local misorientation/strain around cracks and bridges.	High-resolution micro-Raman; EBSD/HR-EBSD on polished sections; TEM diffraction for narrow bridges.	Map both sides of representative cracks and report spatial step size.	Links eigenstrain and crystallography to crack turning/coalescence.
Separation test	Loading mode; fixture/bond; loaded area; rate; full force–displacement curve; peak force; failure location; successful/failed releases.	Normal-tension reference test preferred for comparison.	At least five coupon replicates per condition for screening; report every failure mode.	Makes nominal separation stress interpretable and comparable.
Paired fracture surfaces	Wafer and ingot surface roughness, maximum step height, chemistry/phase at matching regions, subsurface damage depth.	Confocal/AFM; SEM; Raman; EDS/XPS; cross-sectional SEM/TEM.	Register matching locations on both surfaces and state measurement area/filter.	Confirms path fidelity and determines finishing allowance.
Manufacturing metrics	Full-cycle time; material removed from wafer and ingot; intact-release yield; edge damage; post-finish thickness/TTV/bow/warp; number of ingot reuse cycles.	Production log + dimensional metrology.	Report distributions, not only best-case values.	Connects low separation load to true manufacturing benefit.

## Data Availability

No new datasets were generated. Numerical values plotted or tabulated in this review were compiled from the cited publications.
